# Host Control of Symbiont Natural Product Chemistry in Cryptic Populations of the Tunicate *Lissoclinum patella*

**DOI:** 10.1371/journal.pone.0095850

**Published:** 2014-05-02

**Authors:** Jason C. Kwan, Ma. Diarey B. Tianero, Mohamed S. Donia, Thomas P. Wyche, Tim S. Bugni, Eric W. Schmidt

**Affiliations:** 1 Department of Medicinal Chemistry, University of Utah, Salt Lake City, Utah, United States of America; 2 Pharmaceutical Sciences Division, University of Wisconsin–Madison, Madison, Wisconsin, United States of America; University of East Anglia, United Kingdom

## Abstract

Natural products (secondary metabolites) found in marine invertebrates are often thought to be produced by resident symbiotic bacteria, and these products appear to play a major role in the symbiotic interaction of bacteria and their hosts. In these animals, there is extensive variation, both in chemistry and in the symbiotic bacteria that produce them. Here, we sought to answer the question of what factors underlie chemical variation in the ocean. As a model, we investigated the colonial tunicate *Lissoclinum patella* because of its rich and varied chemistry and its broad geographic range. We sequenced mitochondrial cytochrome *c* oxidase 1 (COXI) genes, and found that animals classified as *L. patella* fall into three phylogenetic groups that may encompass several cryptic species. The presence of individual natural products followed the phylogenetic relationship of the host animals, even though the compounds are produced by symbiotic bacteria that do not follow host phylogeny. In sum, we show that cryptic populations of animals underlie the observed chemical diversity, suggesting that the host controls selection for particular secondary metabolite pathways. These results imply novel approaches to obtain chemical diversity from the oceans, and also demonstrate that the diversity of marine natural products may be greatly impacted by cryptic local extinctions.

## Introduction

Secondary metabolites are often bioactive and are thus an attractive source of lead compounds in drug discovery efforts [1], [2]. In many cases, secondary metabolites isolated from higher organisms such as marine invertebrates are thought to ultimately derive from symbiotic bacteria residing in these hosts [3], [4]. In the majority of cases, little is known about the symbionts that produce secondary metabolites due to difficulties in culturing and/or sequencing their genomes directly from complex microbiomes. However, understanding these symbionts' lifestyle is of critical importance, both to natural products discovery and chemical ecology. In our own efforts, we have used the tunicate *Lissoclinum patella* as a model system to understand the interactions between microbial symbionts, host animals and secondary metabolite chemistry.

*L. patella* is a colonial tunicate in the family Didemnidae, with a wide distribution across much of the Western Pacific [5]. Like many didemnid species, *L. patella* harbors a photosynthetic symbiont, the cyanobacterium *Prochloron didemni* [6]. *P. didemni* carries out several metabolic functions for the host [7], and has been shown to synthesize a series of highly modified cyclic ribosomal peptides, termed cyanobactins [8]. The biosynthetic pathways that make cyanobactins are all related to the prototypical patellamide pathway [8]. One or several precursor peptides are expressed, containing the residues that are incorporated into the finished compounds, flanked by recognition sequences and a leader peptide on the N-terminus [9]. A heterocyclase may act on the immature precursor peptide, to produce methyloxazoline, thiazoline and oxazoline from threonine, cysteine and serine, respectively. Optionally, these heterocycles can be oxidized by an oxidase in the pathway (i.e. to methyloxazole, thiazole, oxazole). The leader peptide and the 5′ recognition sequence are then cleaved by a protease homologous to PatA. The last step in the process is the cleavage of the 3′ recognition sequence and macrocylization of the precursor peptide by a PatG homolog. In some pathways, the macrocycle can be further prenylated, if suitable side chains remain. Remarkably, the patellamide pathway and relatives are extremely tolerant to altered precursor cassettes [10], [11], and are capable of processing precursor sequences quite unlike the naturally occuring compounds. It is clear that the currently known cyanobactins account for a miniscule portion of the chemical diversity that is biosynthetically possible, suggesting strong evolutionary or other influences on natural systems.

Beyond *P. didemni*, *L. patella* has a complex microbiome of resident microflora, which contribute to secondary metabolite production [7] and which vary according to microhabitat within the animal [12]. We recently described another symbiont, *Candidatus* Endolissoclinum faulkneri, which is found only in a subset of *L. patella* animals and is associated with the presence of the highly cytotoxic patellazoles [13], that may serve a protective function for the host animal. Our analysis of *Ca.* E. faulkneri genomes [14] indicates that the bacterium is a long-term symbiont that is exclusively vertically transmitted. Strains of *Ca.* E. faulkneri from phylogenetically distant hosts are correspondingly divergent in genomic sequence, indicating genetic isolation [14]. The patellazoles biosynthetic pathway is a large polyketide synthase (PKS) system, which has been maintained for the ∼6–31 million years that *Ca.* E. faulkneri has been associated with *L. patella* [14].

In contrast to *Ca.* E. faulkneri, several lines of evidence suggest that *P. didemni* can be transmitted between hosts both horizontally and vertically. *P. didemni* genomes obtained from geographically distant animals are remarkably similar (above 97% nucleotide sequence identity across the whole genome) [15], indicating that these strains are not genetically isolated. This strongly suggests there is at least a transient free-living fraction of the *P. didemni* population that can move between hosts. Consistent with this notion, although *P. didemni* has never been cultured outside of its host, genome analysis suggests that independent life may be possible [7]. In fact, it has been found that the microenvironment inhabited by *P. didemni* varies significantly in terms of O_2_ saturation and pH during dark/light cycles, indicating that in contrast to reduced-genome intracellular symbionts, *P. didemni* must maintain the ability to adapt to different environmental conditions [16]. Stable *P. didemni* symbiosis is limited to the didemnids, but unstable associations have been reported in other host groups, such as holothurians and sponges [6]. Amongst the didemnids, *P. didemni* phylogeny has been found to be independent of host species, and the distribution of photobionts amongst this group suggests multiple symbiosis establishment events [17]. The presence of a specialized vertical transmission apparatus only in *Diplosoma* likewise suggests parallel evolution of such mechanisms corresponding to multiple origins for this symbiosis [6], [18].

Consistent with findings in *P. didemni*–didemnid relationships, we previously found a random distribution of *P. didemni* secondary metabolite pathways across and within host species [15]. However, we observed that in some cases, different animal species collected in the same vicinity contained different secondary metabolites, suggesting a degree of symbiont selection. We also observed that chemistry is not consistent across all *L. patella* samples. Many ascidian species have been found to encompass cryptic species, due to their similar or identical morphologies [19]–[23]. We hypothesized that similarly, the designation *L. patella* may contain several divergent populations. In the present work, we show that *L. patella* encompasses at least three distinct phylogenetic groups, and that these groups contain different secondary metabolites. In the case of symbionts that can be horizontally-acquired, such as *P. didemni*, this suggests the host is involved in secondary metabolite selection from a free-living pool.

## Results and Discussion

**Figure 1:**
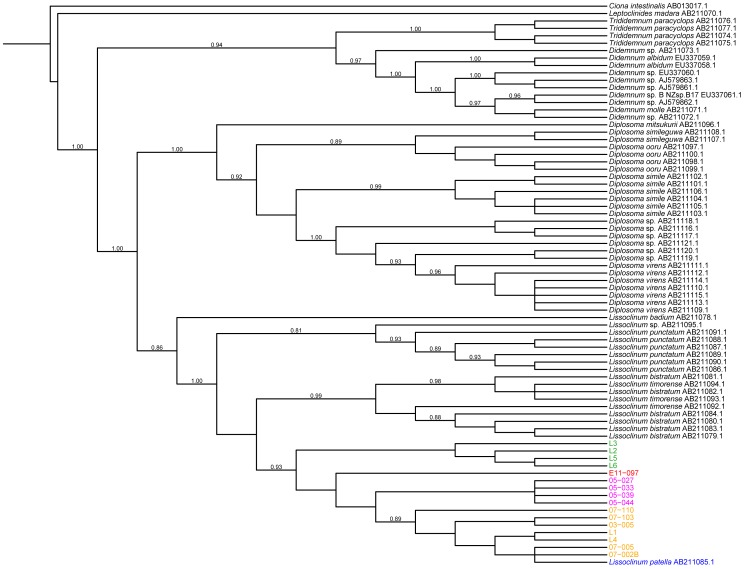
Phylogenetic tree of 18S rRNA nucleotide sequences from our collected *L. patella* animals and other Didemnidae, with *Ciona intestinalis* acting as an outgroup.

**Figure 2:**
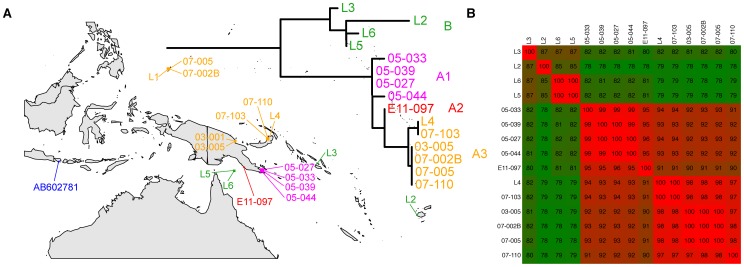
Collection locations, phylogeny and divergence of *Lissoclinum patella* individuals collected across areas of the Southeastern Pacific between 2002 and 2011. (a) Collection sites, with a portion of the phylogenetic tree based on mitochondrial cytochrome c oxidase 1 (COXI) genes overlaid, with individuals colored by clades that diverge by 5% or more in their nucleotide sequence, as shown in (b).

**Figure 3:**
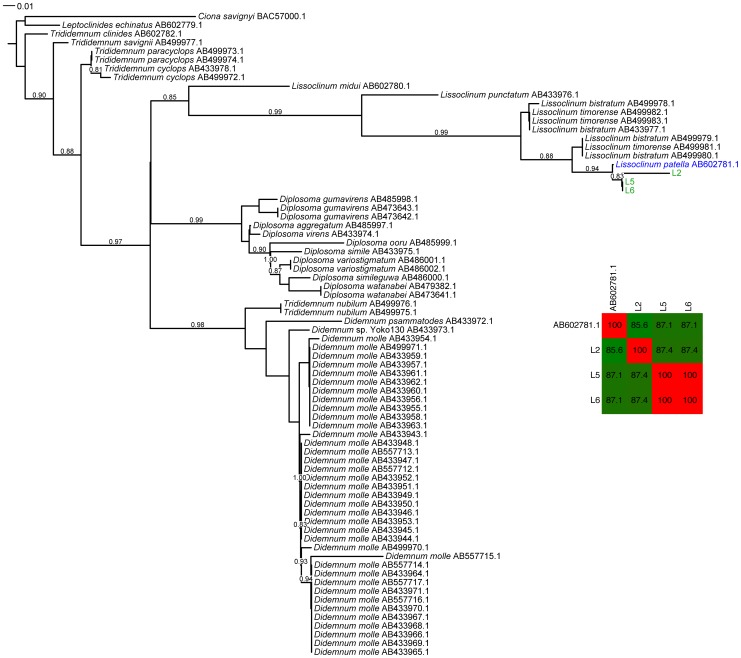
Phylogenetic tree of mitochondrial cytochrome *c* oxidase 1 (COXI) protein sequences from our collected *L. patella* animals and other Didemnidae, with *Ciona savignyi* acting as the outgroup. Note: the *Didemnum vexillum* clade is collapsed for space. The Didemnidae COXI genes found in the NCBI database cover two non-overlapping regions of the gene (see Main Text), and therefore two separate trees were constructed (for the other tree, see Figure 4).

**Figure 4:**
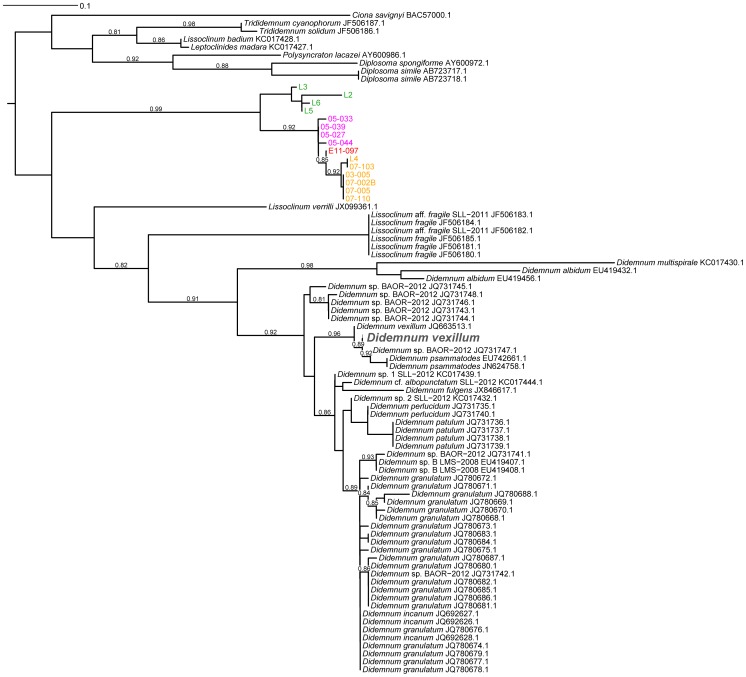
Phylogenetic tree of mitochondrial cytochrome *c* oxidase 1 (COXI) protein sequences from Didemnidae animals not included in Figure S3. The COXI sequences for *L. patella* amimals L2, L5 and L6 used to make this tree were obtained from the respective mitochondrial genome assembly from Illumina sequencing data. The inset identity matrix shows the pairwise nucleotide identities of sequences in this clade, indicating that *L. patella* sequence AB602781.1 likely is a group B animal.

As a result of our long-standing interest in didemnid tunicates, *P. didemni* and the cyanobactins, we have collected specimens of *L. patella* spanning a large geographic area from Fiji to Palau between 2002 and 2011. We were able to amplify host 18S rRNA genes from a number of these samples, and all show >98% nucleotide identity to an 18S rRNA sequence in the NCBI database identified as *L. patella* (accession no. AB211085, see Table S1). We constructed a phylogenetic tree based on these nucleotide sequences plus other members of Didemnidae from the NCBI database and the *Ciona intestinalis* 18S rRNA gene to act as an outgroup. All of our sequences formed a clade along with the type *L. patella* sequence (see Fig. 1). We then turned our attention to the mitochondrial cytochrome *c* oxidase I (COXI) gene, because this marker has been used for fine phylogenetic distinctions [24] and identifying cryptic species, including many types of tunicate [19]–[23]. Using a variety of primer sets (see Table 1), we were able to amplify COXI genes from 15 animals (see Fig. 2(a)). The resulting sequences were pooled with other Didemnidae COXI sequences from NCBI along with a *Ciona savigyni* COXI sequence to act as an outgroup in a tree based on the translated protein sequences. Unfortunately, we found that amongst these NCBI sequences, two non-overlapping sections of the COXI gene were present, and thus we could not make one complete COXI tree with all sequences (see Figs. 3 and 4). In particular, the only reported *L. patella* COXI sequence could not be included in the tree containing our sequences, although we were able to compare it to full length COXI sequences assembled from shotgun Illumina data obtained from three animals (vide infra).

**Table 1:** Primers and probes used in this study.

Primer name	Sequence	Citation	Notes
L_patella_18SF	GCTAAGCCATGCAAGTGCAAG	Kwan *et al.* 2012 [13]	Amplification of *L. patella* 18S rRNA gene
L_patella_18SR	ACGACTTTTACTTCCTCTAAGCGC	Kwan *et al.* 2012 [13]	Amplification of *L. patella* 18S rRNA gene
18S_F3	GATCCTGCCAGTAGTBATAT	Yokobori *et al.* 2006 [17]	Amplification of *L. patella* 18S rRNA gene
18S_R3	TGATCCTTCTGCAGGTTCA	Yokobori *et al.* 2006 [17]	Amplification of *L. patella* 18S rRNA gene
P1P4_COXI_F	GCTTTAGTTAGGACATCAATAAG	Kwan *et al.* 2012 [13]	Amplification of *L. patella* COXI gene
P1P4_COXI_R	ACTAACCACAATACAGGAATATC	Kwan *et al.* 2012 [13]	Amplification of *L. patella* COXI gene
P2P3_COXI_F	GCTTTAATTGGAACATCTATAAG	Kwan *et al.* 2012 [13]	Amplification of *L. patella* COXI gene
P2P3_COXI_R	CAGGAATGTCGAAACGAATG	Kwan *et al.* 2012 [13]	Amplification of *L. patella* COXI gene
Eric_COXI_F	TATRGTTRGKGGTTTTGG	This study	Amplification of *L. patella* COXI gene
Eric_COXI_R	CGGARAAATAAGCTCGWG	This study	Amplification of *L. patella* COXI gene
L_patella_18SF2	AGGCAGAAGAACCACACGAGG	This study	Sequencing of *L. patella* 18S rRNA gene
L_patella_18SR2	GCCACCACGACCATTCGAAAG	This study	Sequencing of *L. patella* 18S rRNA gene
M13_4_TOPO-F	GTAAAACGACGGCCAG	Supplied with TOPO-TA kit	Sequencing primer for TOPO clones
M13_4_TOPO-R	CAGGAAACAGCTATGAC	Supplied with TOPO-TA kit	Sequencing primer for TOPO clones

Our collections of *L. patella* fall into at least three separate clades (see Fig. 2 and Fig. 3). We already observed that animals containing *Ca.* E. faulkneri and patellazoles were restricted to a divergent clade we termed group ‘B’ [13], [14]. Detailed analysis of the COXI nucleotide identities of this clade reveal it contains three highly divergent cryptic populations which could be different species [14]. We now see that the animals of group ‘A’ are also somewhat divergent. When we examined in detail the pairwise nucleotide identities of COXI sequence (see Fig. 2(b)), we saw that animals collected in 2005 from Southeastern Papua New Guinea have significantly diverged from other group ‘A’ animals, with identities ranging from 91–95%. This divergence is on the order of other cases of cryptic speciations claimed in tunicates based on COXI divergences between 2 and 16.5% [19]–[23]. In a survey assessing the use of COXI for phylogenetic distinctions, it was found that conspecific samples rarely diverged more than 2% in nucleotide identity [24]. Therefore it is possible that animals from the ′05 collection are a distinct cryptic species from other group ‘A’ animals. We found evidence that there may be further cryptic populations in the remaining group ‘A’ animals, as animal E11-097 exhibited an intermediate divergence between both the 2005 collection and the remainder of group ‘A’ (90–95% identity). We therefore term these groups as A1 (05–033, 05–039, 05–027, 05–044), A2 (E11-097) and A3 (L4, 07-103, 03-005, 07-002B, 07-005, and 07-110). Interestingly, we were able to compare the *L. patella* COXI sequence from NCBI (AB602781.1) with full length COXI sequences assembled from Illumina shotgun sequencing data of L2, L5 and L6 (see Fig. 4). This revealed that AB602781.1 was as closely related to L5 and L6 as it was to L2. The pairwise sequence identities between all three groups (L5/L6, L2 and AB602781.1) were similarly and significantly different. This suggests that AB602781.1, collected in Sanur, Bali (see Fig. 2), may be another member of group B and could contain *Ca.* E. faulkneri and the patellazoles.

The divergent group B contains individuals collected over a wide swath of the Pacific, from the Eastern Fields region south of Papua New Guinea to Fiji. Conversely, group A1 contains only individuals collected off Southeastern shores of Papua New Guinea and is minimally divergent. These results alone would suggest that a primary influence on *L. patella* phylogeny is geography along with founder effects, similar to some populations of sponges [25], [26]. However, group A3 is phylogenetically quite uniform (see Fig. 2) and yet covers a large area encompassing the Bismarck Sea and Palau. Further sampling is required to determine whether A1 and A2 are truly more geographically restricted than A3, and this will likely reveal the full extent of these groups' ranges and also determine whether any coexist in the same locale. Coexistence might suggest that there is little genetic exchange between the groups; for instance there are several color morphotypes of the didemnid tunicate *Didemnum molle* that are phylogenetically distinct [27]. Sometimes several morphotypes occupy the same area and thus there may be mechanisms to maintain reproductive isolation of these forms. From analysis of the aligned COXI sequences in our tree set, the different *D. molle* morphotypes shared between 89 and 97% nucleotide identity (see Table S2). Another didemnid tunicate, *D. vexillum*, was recently found to be composed of two phylogenetic clades [28]. The two clades share ∼96% COXI nucleotide identity (see Table S2), and the authors concluded that these were not separate species. Nevertheless, colony fusion experiments showed significantly higher success rates amongst the genetically similar invasive form from New Zealand versus the genetically diverse population from Japan [28].

**Figure 5:**
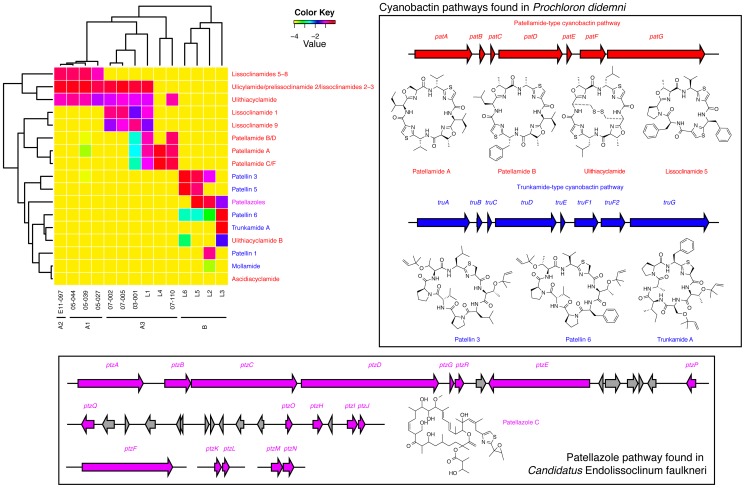
Heatmap and hierarchical clustering of select secondary metabolite peak volumes in LCMS runs on *L. patella* extracts (top left). Clustering based on secondary metabolites that are produced by the symbiotic bacteria *Prochloron didemni* and *Ca.* Endolissoclinum faulkneri closely follows the hosts' phylogeny as determined by COXI sequences (see Fig. 2). The *P. didemni* compounds shown are all cyanobactins produced either by a patellamide-type pathway (red), or a trunkamide-type pathway (blue). These two types of are closely related ribosomal pathways that are highly tolerant to changes in the precursor peptide sequence. The patellazoles (magenta) are produced by another symbiont, *Candidatus* Endolissoclinum faulkneri, by a polyketide synthase pathway.

We then examined the secondary metabolite chemistry of *L. patella* animals by LCMS, identifying known cyanobactins and patellazoles previously found in *L. patella* based on their mass. We used skiff, a Perl script used in the Clovr-16S pipeline [29], [30] to analyze the tabulated peak areas. This script's intended purpose is to take tables of 16S abundances, binned at an arbitrary taxonomic level, and create a heatmap and dendrograms that reflect the Euclidean distance between normalized samples and bins. Because skiff is agnostic as to the type of data it receives, we were able to use it to visualize the Euclidean distance between samples based on *L. patella* chemistry as shown by a dendrogram (see Fig. 5). With this method, we observe that the secondary metabolites present mirror the clades we determined in our COXI phylogenetic tree, except that E11-097 is closer chemically to the A1 clade than phylogeny would suggest. The A1 members are classified by the presence of lissoclinamides 5–8, ulicyclamide and isomers, as well as ulithiacyclamide. The A3 group lack lissoclinamides 5–8 and in addition can contain lissoclinamide 1, 9 and patellamides. The B group are quite chemically distinct. As well as containing the patellazoles, they also contained different cyanobactins compared to group A animals, including the prenylated patellins and trunkamide A.

It is not surprising that the patellazoles are limited to group B animals [13], [14], because these compounds are produced by an exclusively vertically transmitted symbiont that is not found in other clades (vide supra). However, it is notable that even though *P. didemni* populations are highly uniform and do not correlate with host phylogeny [6], there is a strong host phylogenetic signal for their secondary metabolites. We previously found a high sequence identity across three whole *P. didemni* genomes (>97%), in both patellazole containing and patellazole-negative animals [15]. This suggests that *P. didemni* strains are not genetically isolated, and that there must be significant horizontal exchange between strains in different hosts, in addition to well-established vertical transmission mechanisms [18]. The P2 and P3 *P. didemni* genomes come from animals we now know are significantly divergent (L2 and L3, respectively). The P1 genome comes from animal L1. The majority of the extracted DNA from this animal was used in the extensive sequencing of the P1 genome [7], [15], and therefore we were not able to obtain a COXI sequence, but chemically it aligns to members of group A3 and was collected from the same geographic area as other members of this group. We show that although P1 and P2/P3 have highly similar genomes, they produce different secondary metabolites. Together, our results suggest that there is some degree of selection for *P. didemni* strains based on secondary metabolism, and that this correlates with host phylogeny. The patellazoles are highly toxic and therefore likely to be defensive in function. This may also be the case for the cyanobactins, although they are not generally as cytotoxic as the patellazoles. Some have suggested that cyanobactins may have metal binding capabilities, and some have moderate cytotoxicity [31], but their true ecological function remains obscure. We have found, however, that their distribution is not random, and this may be used as a basis for further exploration of their function. Previous reports of natural product isolation may indicate the potential ranges for the different *L. patella* clades. For instance, patellazoles were previously isolated from an animal collected in Guam [32], and compounds related to both trunkamides [33] and patellamides [34] have been isolated from animals collected in the Great Barrier Reef.

**Figure 6:**
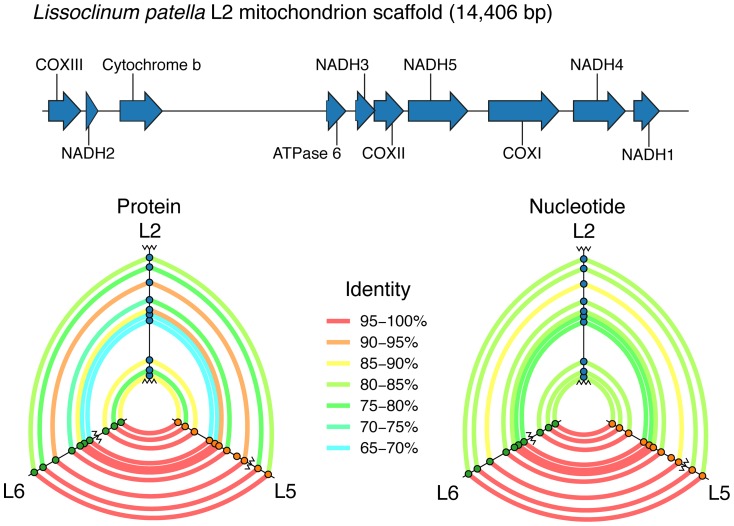
Schematic representation of the draft mitochondrial genome of *L. patella* animal L2 (top, to scale). The hive plots [61] at the bottom of the figure show that the L2 assembly is syntenic with contigs assembled of the mitochondrial genomes of L5 and L6. In these plots, protein-coding genes are represented as circles, and contig boundaries are represented as zigzag lines. Homologous genes are joined by curved lines colored according to the sequence identity of the gene relevant gene pair. Abbreviations: COX, cytochrome *c* oxidase; NADH, nicotinamide adenine dinucleotide dehydrogenase.

The mitochondrion is thought to be the result of an ancient endosymbiosis event in the early evolution of eukaryotes [35], and its tiny genome is therefore likely the end result of the process of genome degradation and erosion observed in endosymbiotic bacteria [36]. Like more recent endosymbionts, mitochondia exhibit accelerated evolution because of their population structure and lack of DNA repair pathways [36], and their genome sequences can be used to infer a great deal about the hosts' evolutionary history. Additionally, in tunicates it has been shown that gene order in mitochondria is hypervariable, potentially providing an additional phylogenetic signal [37]. We had previously obtained shotgun metagenomic sequence in Illumina HiSeq runs for three group B animals (L2, L5 and L6, see Materials and Methods), and set out to assemble mitochondrial genomes from them. In all cases, contigs that appeared mitochondrial were high coverage (several hundred × or more), and could be separated from other genomes on this basis. In all cases, mitochondrial assemblies were resolved to single contigs, ranging in size from 12,562 bp (L6) to 14,403 bp (L2), and all were ∼21% GC. Annotated ORFs all correspond to genes previously found in other tunicate genomes [37]–[45] (see Fig. 6), but some genes commonly found were missing from the assemblies (NADH dehydrogenase subunit 4L was missing from L2 and L6, while NADH dehydrogenase subunit 6 and ATPase F0 subunit 8 were not found in any of the assemblies). This may be due to general difficulties in assembling such high-AT sequence. The L2 mitochondrion assembly contained an unannotated section, roughly equivalent in size to the small and large subunit rRNA genes in other tunicate mitochondria. This section may include the L2 mitochondrial rRNA genes, but only small parts of the sequence showed any homology to tunicate mitochondrial rRNA genes in BLASTN searches against the NCBI database. Independent assembly efforts in L5 and L6 were syntenic with the L2 assembly (see Fig. 6), but they appear to lack the putative rRNA region in L2. The consistent synteny across three samples suggests that there are no misassembled portions of the L2 assembly. As with previously reported tunicate mitochondrial genomes [37]–[45], all genes are on the same strand, but the gene order exhibited is unique. We previously determined that the members of group B may represent several cryptic species, with COXI identities suggesting that animals L2 and L5 diverged somewhere between 6 and 31 million years ago [14]. As expected, L5 and L6 share greater gene identities with each other than either one has with L2 (see Fig. 6). Interestingly, our analysis shows that L5 and L6 are not clones. These animals were collected in the same vicinity (within ∼100 m); while L5 contains *Ca.* E. faulkneri and patellazoles, L6 has lost *Ca.* E. faulkneri and contains a potentially pathogenic bacterium in its place that we termed *Ca.* Xenolissoclinum pacificiensis [14]. Because L5 and L6 have highly similar mitochondrial genomes, colony fusion may be possible [28], and therefore we cannot exclude the possibility that the loss of *Ca.* E. faulkneri in L6 is reversible through this mechanism. Further studies will be needed in order to investigate the structure of this population, and whether the loss of *Ca.* E. faulkneri in L6 is recent and/or stable. Such studies might be a unique opportunity to investigate the influence of symbionts and natural products on host population structure and speciation.

Variation in the distribution and abundance of natural products is a significant problem that affects the utility of natural compounds in drug discovery efforts. Often vanishingly small amounts of a compound are isolated in an initial collection. Although only small amounts are required for the characterization of structure and *in vitro* activity, recollection for further development is often challenging. Our efforts here show that one of the factors that can control chemical variation in symbiotic systems is unappreciated cryptic speciation of the host, even when symbionts may be obtained from the environment. Similar issues are beginning to be recognized in other secondary metabolite producers. For instance cyanobacteria designated as *Lyngbya majuscula* have been credited with several hundred natural products in the literature [46], [47]. Recent genomic sequencing of a strain that fell under this classification (now *Moorea producta* [46]) suggests *Lyngbya majuscula* could be a morphologically identical species complex whose natural products may be a marker of phylogeny.

Our mitochondrial genome sequences hint at the complexity of symbiotic interactions in the environment, where individual hosts can be found that have lost even stable symbionts. Loss and gain of strictly vertical symbionts may play a major role in host speciation if these events affect fitness and reproductive compatibility [48], consistent with previous models of symbiosis. We observed one such local extinction in animal L6, which lacks *Ca.* E. faulkneri and is likely unable to regain this symbiont, potentially driving host speciation and adaptation in L6's descendents.

This study shows that the previously supposed random distribution of *P. didemni*–produced cyanobactins is in fact based on host phylogeny. Genomic data indicate that this symbiont is highly uniform, and therefore populations within individual hosts are *not* genetically isolated and must undergo frequent horizontal exchange between didemnid hosts. Thus, our results implicate host-dependent recruitment of *P. didemni* based on secondary metabolite production. Because cyanobactin pathways are highly tolerant to precursor peptide mutations [11], [49], the host may play a major role in maintaining ecologically important precursor sequences. These results have implications for biodiversity and drug discovery. In tandem with previous results showing new compounds can be isolated by surveying individual ascidian colonies [15], [50], it is now clear that cryptic populations of ascidians are an untapped source of new potential pharmaceuticals. In turn, if a local, cryptic population goes extinct due to habitat loss, there is a likelihood that potential pharmaceuticals will be permanently lost to science. While extinction of cryptic species is often proposed to negatively impact drug discovery, there is a prevalent contrary view that, at least among bacteria, ‘everything is everywhere’. Here we provide a concrete example of how important bacterial compounds might be lost through destruction of local habitats.

## Materials and Methods

### Extraction of DNA and sequencing of phylogenetic markers

Permission to perform field research was granted by the Papua New Guinea Department of Environment and Conservation, the governments of Palau, Fiji and Solomon Islands. These efforts were facilitated by the University of Papua New Guinea, the Coral Reef Research Foundation and the University of South Pacific, respectively. Samples of *L. patella* were collected from the sites indicated in Figure 2 and preserved in RNAlater. Portions of each sample were set aside as frozen specimens for chemical analysis. DNA was extracted from tunicate samples preserved in RNAlater either using an established tunicate method [51], the Qiagen DNeasy kit, or by direct pulverizing of tissue in DMSO. Tunicate 18S rRNA and mitochondrial COXI genes were amplified using primers shown in Table 1, with Platinum Taq High Fidelity (Invitrogen). In all cases, PCR reactions were 10 µL volume, containing 0.1 µL Taq, 1× of the supplied buffer, 2 µM each primer, 0.2 mM each dNTP (Invitrogen) and 2.0 mM MgSO_4_. Reactions consisted of hot start (94°C, 2 min), followed by 35 cycles of [94°C/30 s, variable annealing temperature/30 s, 68°C/1 min per kb extension (minimum 45 s)], then a final extension step of 68°C/10 min. PCR products were either Sanger sequenced directly with the relevant primers, or else cloned using the Topo-TA cloning kit (Invitrogen) before sequencing.

### Construction of phylogenetic trees and identity matrices

Marker sequences (both 18S rRNA and COXI nucleotide sequences) for members of the family Didemnidae and *Ciona intestinalis* or *Ciona savignyi* were downloaded from the NCBI database. The *Ciona* sequences acted as outgroups for rooting the trees. Database 18S rRNA sequences, along with experimental sequences from *L. patella* samples were aligned with Clustal Omega [52]. The alignment was inspected manually in ClustalX [53], and sequences that were extremely short or unilaterally introduced large inserts were discarded. The alignment was trimmed using a Perl script (tim_aligned_fasta.pl [13]), and used to construct a phylogenetic tree with FastTreeMP [54] with the parameters -slow -spr 5 -mlacc 3 -gamma -gtr -nt. All trees were viewed and manipulated using the Interactive Tree of Life server [55].

COXI sequences were translated into protein sequences using the ascidian mitochondrial translation table (NCBI translation table 13), then aligned with Clustal Omega. It was found that COXI sequences from NCBI aligned to two distinct, non-overlapping regions of the *Ciona savignyi* sequence (accession no. BAC57000.1), and so two distinct trees were constructed (Figures 3 and 4), one of which included sections of COXI sequence obtained from the assembled mitochondrial genomes of L2, L5 and L6 (vide infra). In each case the alignments were manually inspected and trimmed as with the 18S rRNA alignment, before the trees were constructed with FastTreeMP using the parameters -slow -spr 10 -mlacc 3 -bionj -gamma. In order to determine the nucleotide identities of the sequences in the trees, the original nucleotide sequences were aligned to the protein alignments with a Perl script (nucleotide_translation_alignment_2.pl, Text S1). Pairwise identities were calculated from this alignment with another Perl script (identity_matrix.pl [13]).

### UHPLC/HRMS analysis

Frozen chemistry voucher samples of each animal were freeze-dried, then sequentially extracted with chloroform and methanol. The combined extracts were dried down and passed over a small C_18_ plug, eluting with methanol. LC/MS data were acquired using a Bruker MaXis ESI-Q-TOF mass spectrometer coupled with a Waters Acquity UPLC system operated by Bruker Hystar software. A gradient of MeOH and H_2_O (containing 0.1% formic acid) was used with a flow rate of 0.3 mL/min on a RP C18 column (Phenomenex Kinetex 2.6 µm, 2.1 mm × 100 mm). The gradient went from 10% MeOH/90% H_2_O to 97% MeOH/3% H_2_O in 12 mins, followed by 97% MeOH/3% H_2_O held for 3.5 mins. Full scan mass spectra (*m*/*z* 150-1550) were measured in positive ESI mode. The mass spectrometer was operated using previously published parameters [56]. Tune mix (Agilent, ESI-L low concentration) was introduced through a divert valve at the end of each chromatographic run for automatic internal calibration.

### Construction of secondary metabolite heatmap and dendrogram (Figure 5)

Raw data files from LCMS runs were converted to mzXML format and processed in MZMine [57] according to the following procedure: 1. peak detection in centroid mode with a noise level cutoff of 5.0 × 103; 2. chromatogram building with a minimum time span of 0.05 min, minimum peak height of 2.5, and 5.0 ppm *m*/*z* tolerance; 3. chromatogram deconvolution using the local minimum search algorithm, a chromatogram threshold of 65.0%, a search minimum retention time range of 0.05 min, a minimum relative height of 5.0%, a minimum absolute height of 5.0 × 103, and a minimum ratio of peak top/edge of 2; 5. isotopic peak grouping with a *m*/*z* tolerance of 5.0 ppm, retention time tolerance of 0.01 min, maximum charge of +2, assuming monoisotopic shape with the lowest *m*/*z* being representative; 6. peaks list row filtering with a minimum of 1 peak in a row, a minimum of 1 peak in an isotopic pattern, a peak duration range of 0.0–2.0 min and auto *m*/*z* and retention time. Compounds were identified in MZMine using a custom database containing compounds previously found in *L. patella*, with a tolerance of 5 ppm error (see Text S2). The peak areas reported in MZMine were tabulated, and compounds arising from the same biosynthetic precursor peptides or pathways were added together (including both [M + H]+ and [M + Na]+ ions). The table was used as an input for skiff (http://clovr.org/docs/skiff/), a Perl script that is a component of the CloVR-16S pipeline [29], [30]. This script normally takes tables of 16S abundances for groups of samples, binned according to a certain level of phylogenetic classification. In this case, the peak areas were expressed as a fraction of the sum of assigned peak areas for each sample, and the log_10_ of each fraction was used to plot the heatmap. Clustering of samples (i.e. the dendrogram portion of Figure 5) was achieved by calculating the Euclidean distance between samples based on these transformed values.

### Sequencing, assembly, annotation and comparison of draft ascidian mitochondrial genomes

Assemblies of the mitochondrial genomes of *L. patella* animals L2, L5 and L6 were constructed from Illumina HiSeq 2000 datasets previously reported [13], [14]. In each case, 10% of the full dataset was used (8.0 M paired reads for L2, 14.8 M paired reads for L5 and 20.0 M paired reads for L6). A script was used to screen out PCR duplicates (https://github.com/ibest/GRC_Scripts/blob/master/screen_duplicates_PE.py), then the reads were filtered for length > 40 bp and quality > 30 with Seqyclean (https://bitbucket.org/izhbannikov/seqyclean). Only the first 4.8 M filtered reads were used for subsequent processing in L6, due to high mitochondial genome coverage. Overlapping paired reads were then joined with FLASH [58], then both paired and overlapping reads were subjected to assembly with SPAdes [59] in -careful mode. All three mitochondrial genomes were resolved into a single contig, using K-mer values of 67,73,77,83,87 (L2); 41,45,51,55,61 (L5); and 77 (L6). The genomes were annotated manually in Artemis [60], and found to all be syntenic (although with different break points in the respective contigs). Sequence comparisons were carried out by first aligning gene protein sequences with ClustalX and then constructing nucleotide alignments from these as described above. The hive plots [61] that form part of Figure 6 were created using the D3JS javascript framework (http://d3js.org), by adaptation of an example plot (http://bl.ocks.org/mbostock/2066415).

### Accession Numbers

The ascidian marker sequences and mitochondrial assemblies have been submitted to the National Center for Biotechnology Information (NCBI) (http://www.ncbi.nlm.nih.gov). The accession numbers are as follows. **18S rRNA genes**: L3, KJ009375; L2, KJ009376; L5, KJ009377; L6, KJ009378; E11-097, KJ009379; 05-027, KJ009380; 05-033, KJ009381; 05-039, KJ009382; 05-044, KJ009383; 07-110, KJ009384; 07-103, KJ009385; 03-005, KJ009386; L1, KJ009387; L4, KJ009388; 07-005, KJ009389; 07-002B, KJ009390. **COXI genes**: L3, KJ009363; 05-033, KJ009364; 05-039, KJ009365; 05-027, KJ009366; 05-044, KJ009367; E11-097, KJ009368; L4, KJ009369; 07-103, KJ009370; 03-005, KJ009371; 07-002B, KJ009372; 07-005, KJ009373; 07-110, KJ009374. **Mitochondrial genome assemblies**: L2, KJ596321; L5, KJ596322; L6, KJ596323.

## Supporting Information

Table S1 Nucleotide identities of *L. patella* 18S rRNA genes used to construct the tree in Figure 1. Found at DOI: xxxxxxx (25K XLSX).
(XLSX)

Table S2 Nucleotide identities of *D. molle* and *D. vexillum* COXI genes used to construct the trees in Figures 3 and 4. Found at DOI: xxxxxxx (48K XLSX).
(XLSX)

Text S1 **Perl source code for nucleotide_translation_alignment_2.pl. Found at DOI: xxxxxxx (2.6K PL).**
(PL) Click here for additional data file.

Text S2 **Custom cyanobactin and patellazoles database used to identify LCMS peaks in MZmine. Found at DOI: xxxxxxx (3.7K CSV).**
(CSV)
