# Correction: Host Control of Symbiont Natural Product Chemistry in Cryptic Populations of the Tunicate *Lissoclinum patella*

**DOI:** 10.1371/journal.pone.0106170

**Published:** 2014-08-13

**Authors:** The PLOS ONE Staff

There are errors in the legends for the Supporting Information files Table S1, Table S2, Text S1, and Text S2. Please see the correct legends for Table S1, Table S2, Text S1, and Text S2 here.

## Supporting Information

Table S1 **Nucleotide identities of *L. patella* 18S rRNA genes used to construct the tree in Figure 1.**
(XLSX)

Table S2 **Nucleotide identities of *D. molle* and *D. vexillum* COXI genes used to construct the trees in Figures 3 and 4.**
(XLSX)

Text S1 **Perl source code for nucleotide_translation_alignment_2.pl.**
(PL) Click here for additional data file.

Text S2 **Custom cyanobactin and patellazoles database used to identify LCMS peaks in MZmine.**
(CSV)
